# Global landscape of clinical trials for endometriosis: dynamic trends and future directions

**DOI:** 10.1097/JS9.0000000000002854

**Published:** 2025-06-24

**Authors:** Sijie He, Houhong Li, Li Wan, Xianya Qin

**Affiliations:** Department of Pharmacy, Maternal and Child Health Hospital of Hubei Province, Tongji Medical College, Huazhong University of Science and Technology, Wuhan, China

Endometriosis is a chronic, estrogen-dependent gynecological disorder characterized by ectopic implantation and growth of endometrial-like tissue outside the uterine cavity, commonly associated with chronic pelvic pain, infertility, and impaired quality of life^[1]^. Approximately 5%–10% of reproductive-aged women worldwide are affected, making endometriosis a substantial global public health concern^[1]^. Current treatment options, predominantly hormonal therapies, and surgical interventions remain suboptimal due to frequent recurrence and considerable side effects, highlighting an urgent need for more targeted and effective therapeutic alternatives. Consequently, clinical trials investigating novel pharmacologic interventions are critical for advancing therapeutic strategies.

The clinical trial landscape for endometriosis provides critical insights into emerging therapeutic trends, regional disparities, and unmet needs in drug development. The Informa database is a reliable and authoritative pharmaceutical research resource that serves as a comprehensive online trial repository, incorporating data from ClinicalTrials.gov alongside more than 58,000 additional global sources. To systematically identify high-quality clinical trials focusing on pharmacological interventions for endometriosis, we searched the Informa database using Medical Subject Headings (MeSH) term “Disease is Genitourinary: Endometriosis.” As of April 7, 2025, after excluding observational studies, a total of 744 interventional pharmaceutical clinical trials were included for final analysis (Fig. 1). Notably, this study was conducted in full compliance with the TITAN Guidelines for transparent reporting of artificial intelligence^[2]^.

**Figure 1.**
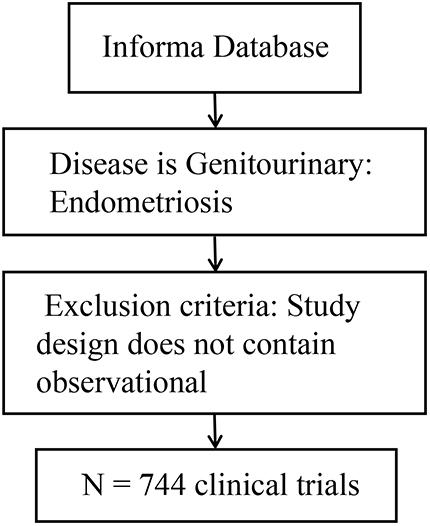
Flowchart.

As illustrated in Figure 2A, the volume of clinical trials focused on endometriosis has steadily increased, particularly reflecting heightened global attention from both medical and pharmaceutical sectors to address significant unmet therapeutic needs in this field, such as managing progesterone resistance, reducing side effects of GnRH antagonists, and developing non-hormonal therapies for chronic pain^[1]^. In particular, the number of early-stage clinical trials (Phases I and II) has risen dramatically, revealing an increase in innovative drug development in this field. Global endometriosis clinical trials exhibit a bimodal dominance by China and the United States, reflecting their strengths in patient populations, research investment, and market potential (Fig. 2B). Japan, Italy, and Germany constitute the second-tier research contributors, with a good clinical research foundation, which continues to promote innovation and new drug development. The Asia-Pacific, Latin America, and Africa regions still have great potential and room for improvement, and are expected to have more research investment and clinical resources integration in the future under the background of globalization. Figure 2C provides an overview of the completion status of the trials. Most clinical trials investigating treatments for endometriosis have progressed smoothly, generating reliable clinical evidence that significantly contributes to therapeutic guidelines and clinical practices. The early termination of a certain percentage of trials is a cause for concern, suggesting that future research should focus on risk management, efficacy prediction, and biomarker development to reduce the risk of trial failure. Figure 2D illustrates the distribution of clinical trial sponsors. The pharmaceutical industry predominates, with active involvement from both large multinational corporations and smaller pharmaceutical enterprises, underscoring significant market potential and commercial interest in endometriosis treatments. Academic institutions mainly promote cutting-edge exploration and basic innovation research, which is gradually translated to the industry. A more effective academic-industry cooperation model may be needed in the future to promote the in-depth development of personalized precision medicine. Clinical trials for endometriosis primarily focused on traditional hormonal pathways, including progesterone receptors, GnRH analogs, and estrogen receptors (Fig. 2E). A considerable proportion of trials involve drugs with unspecified or multi-target mechanisms, reflecting the complexity of endometriosis pathogenesis. The field of endometriosis clinical research is still dominated by hormonal drugs, but also shows a trend towards natural products, non-hormonal drugs, and pain management (Fig. 2F). The prominence of natural products particularly highlights increasing market demand and clinical interest in safer, more tolerable long-term treatment options.

**HIGHLIGHTS**

- The number of early-stage clinical trials (Phases I and II) has risen dramatically, revealing an increase in innovative drug development in this field.
- China and the U.S. lead global trials, while Africa and Latin America lag behind.
- Hormonal therapies (GnRH antagonists) dominate despite adverse bone-density effects.
- Non-hormonal agents targeting P2X3 receptors show limited efficacy in pain relief.
- Preclinical studies continue to explore the underlying pathogenesis of endometriosis.

**Figure 2.**
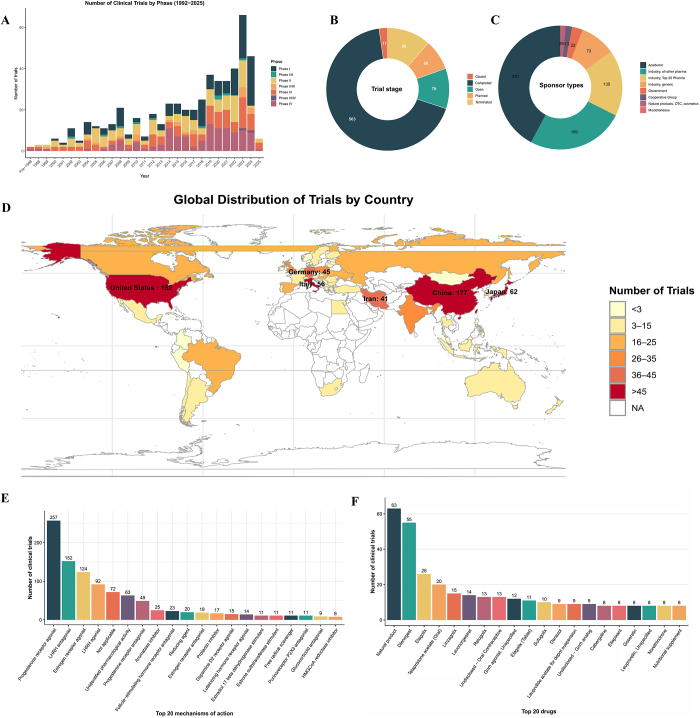
Clinical trials landscape for endometriosis. (A) Trial phase distribution of clinical trials on endometriosis by start years. (B) The distribution clinical trials by trial stage. (C) The distribution clinical trials by sponsor types. (D) Global distribution of trials by country. (E) Top 20 mechanisms of action. (F) Top 20 drugs of action. LHRH, luteinizing hormone-releasing hormone; HMGCoA, 3-hydroxy-3-methyl glutaryl coenzyme A.

The current clinical trial landscape underscores the dominance of hormonal-based interventions, particularly gonadotropin-releasing hormone (GnRH) antagonists such as elagolix, relugolix, and linzagolix, which offer improved side-effect profiles and oral convenience compared to traditional GnRH agonists. Elagolix, the first GnRH antagonist approved for moderate-to-severe endometriosis-associated pain, has been evaluated in an ongoing 48-month Phase 3 trial to assess long-term efficacy, safety, and bone density outcomes with add-back therapy (ABT) versus placebo, where its use with ABT significantly improved dysmenorrhea, non-menstrual pelvic pain, and fatigue for up to 12 months, although it resulted in greater bone mineral density loss compared to placebo^[3]^. An open-label extension of SPIRIT 1/2 showed combination therapy (relugolix 40 mg, estradiol 1 mg, norethindrone acetate 0.5 mg) sustained pain relief over 104 weeks. Although initial bone mineral density slightly decreased, it subsequently stabilized with continued therapy^[4]^. Linzagolix has recently gained attention due to positive results from the phase 3 EDELWEISS 3 trial. Treatment combining linzagolix 200 mg with estradiol 1 mg and norethindrone acetate 0.5 mg significantly alleviated dysmenorrhea and non-menstrual pelvic pain after 3 months. While monotherapy with linzagolix 75 mg significantly reduced dysmenorrhea only^[5]^. Despite these therapeutic advancements, significant challenges persist, particularly regarding bone mineral density loss and limited long-term tolerability, emphasizing the necessity for treatments with minimized systemic hormonal disruption.

Emerging non-hormonal candidates, particularly P2X3 receptor antagonists, although initially promising, have demonstrated limited efficacy in recent clinical evaluations, highlighting the complexity of pain management in endometriosis. Specifically, the P2X3 antagonist eliapixant demonstrated no statistically significant or clinically meaningful improvement in endometriosis-associated pelvic pain compared to placebo in the NCT04614246 trial^[6]^. Similarly, results from the NCT03654326 trial indicated that gefapixant (45 mg twice daily), another P2X3 receptor antagonist, failed to provide superior pain relief over placebo^[7]^.

Preclinical studies are advancing our understanding of endometriosis pathogenesis. Recent findings demonstrate that ectopic endometrial mesenchymal stromal cells activate nearby ovarian stromal cells through the WNT5A pathway, thereby initiating abnormal cellular proliferation and inflammatory responses^[8]^. This highlights WNT5A as a potential target for new diagnostic and therapeutic strategies. Additionally, using the artificial intelligence-based platform PandaOmics, researchers identified GBP2 and HCK as candidate targets, linking immune pathways to endometriosis^[9]^. Investigating immune pathways offers the possibility of developing more precise and effective therapeutic interventions, potentially reducing lesion growth and improving clinical outcomes for patients.

Our analysis is limited by clinical trial registries’ biases, with inconsistent study registration and updates. Furthermore, multi-drug trials pose difficulties for classification and data analysis due to the complexity involved in distinguishing individual drug effects. Advanced statistical methods and subgroup analyses in future research could help elucidate the individual contribution of each drug within combination therapies.

In conclusion, the expanding and increasingly diverse clinical trial landscape holds significant promise for advancing endometriosis management. Future research should emphasize further elucidation of endometriosis pathogenesis to discover novel therapeutic strategies that effectively control disease symptoms while minimizing adverse effects.

## Data Availability

The datasets generated and analyzed during the current study are available in the INFORMA database (https://pharma.id.informa.com/).

## Research registration unique identifying number (UIN)

Our study is a secondary analysis of clinical trial data obtained from the INFORMA database and does not involve direct interaction with human participants or the collection of new data.
